# Cost-effectiveness of the non-pneumatic anti-shock garment (NASG): evidence from a cluster randomized controlled trial in Zambia and Zimbabwe

**DOI:** 10.1186/s12913-015-0694-6

**Published:** 2015-01-28

**Authors:** Janelle Downing, Alison El Ayadi, Suellen Miller, Elizabeth Butrick, Gricelia Mkumba, Thulani Magwali, Christine Kaseba-Sata, James G Kahn

**Affiliations:** Health Services and Policy Analysis, University of California, Berkeley, CA USA; Department of Obstetrics, Gynecology & Reproductive Sciences, Bixby Center for Global Reproductive Health and Policy, University of California, San Francisco, CA USA; Department of Obstetrics and Gynecology, University Teaching Hospital and University of Zambia, Lusaka, Zambia; Department of Obstetrics and Gynecology, University of Zimbabwe, Harare, Zimbabwe; Philip R. Lee Institute for Health Policy Studies and Global Health Sciences, University of California, San Francisco, CA USA

**Keywords:** Maternal mortality, Obstetric hemorrhage, Hypovolemic shock, Non-pneumatic anti-shock garment, NASG, Cost-effectiveness

## Abstract

**Background:**

Obstetric hemorrhage is the leading cause of maternal mortality, particularly in low resource settings where delays in obtaining definitive care contribute to high rates of death. The non-pneumatic anti-shock garment (NASG) first-aid device has been demonstrated to be highly cost-effective when applied at the referral hospital (RH) level. In this analysis we evaluate the incremental cost-effectiveness of early NASG application at the Primary Health Center (PHC) compared to later application at the RH in Zambia and Zimbabwe.

**Methods:**

We obtained data on health outcomes and costs from a cluster-randomized clinical trial (CRCT) and participating study hospitals. We translated health outcomes into disability-adjusted life years (DALYs) using standard methods. Econometric regressions estimated the contribution of earlier PHC NASG application to DALYs and costs, varying geographic covariates (country, referral hospital) to yield regression models best fit to the data. We calculated cost-effectiveness as the ratio of added costs to averted DALYs for earlier PHC NASG application compared to later RH NASG application.

**Results:**

Overall, the cost-effectiveness of early application of the NASG at the primary health care level compared to waiting until arrival at the referral hospital was $21.78 per DALY averted ($15.51 in added costs divided by 0.712 DALYs averted per woman, both statistically significant). By country, the results were very similar in Zambia, though not statistically significant in Zimbabwe. Sensitivity analysis suggests that results are robust to a per-protocol outcome analysis and are sensitive to the cost of blood transfusions.

**Conclusions:**

Early NASG application at the PHC for women in hypovolemic shock has the potential to be cost-effective across many clinical settings. The NASG is designed to reverse shock and decrease further bleeding for women with obstetric hemorrhage; therefore, women who have received the NASG earlier may be better able to survive delays in reaching definitive care at the RH and recover more quickly from shock, all at a cost that is highly acceptable.

## Background

Ninety-nine percent of the 800 maternal deaths that occur each day are in developing countries [1]. While global maternal mortality has been nearly halved from 1990 to 2008, the proportion of maternal deaths in Sub-Saharan Africa has doubled over the same period [1]. For each woman who dies, an estimated 20–30 women survive with morbidities, including infertility, anemia, and depression [2].

Obstetric hemorrhage continues to be the leading cause of maternal mortality and morbidity worldwideC [3]. Uterine atony, the failure of the uterus to contract after delivery, accounts for a majority of post-partum hemorrhage (PPH) cases [3]. Delays in transport, diagnosis and adequate treatment of women experiencing severe hypovolemic shock due to obstetric hemorrhage are common in settings where access to resources and care are limited [4].

**Figure 1:**
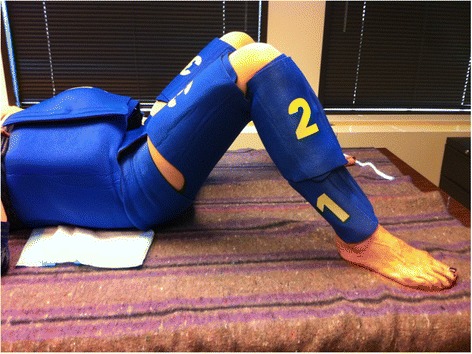
**Image of a non**-**pneumatic anti**-**shock garment (NASG) on a patient.**

The non-pneumatic anti-shock garment (NASG) has been studied as a means to stabilize women with hypovolemic shock secondary to obstetric hemorrhage [5-7]. The NASG is a neoprene compression device that reverses shock by delivering circumferential counter-pressure to the lower body, legs, pelvis, and abdomen (see Figure 1) and decreases blood loss. Use of the garment as a first-aid, temporizing device can reduce the impact of delays in reaching definitive care [8].

Several policy initiatives, such as expanding access to emergency obstetric care, have been established over the past few decades to improve maternal health, yet the evidence base on how to implement these policies and strategies at the health system level remains weak [9]. Scalable health interventions, those that are effective and efficacious when applied to the larger population, are critical in reducing maternal mortalities and morbidities [10]. Information on cost-effectiveness is crucial in planning scale-up and impact of maternal health interventions, but is often lacking [10-12].

The NASG is considered a cost-effective intervention for referral hospitals (RH), based on quasi-experimental studies in Egypt and Nigeria [13]. For women in severe shock, with a mean arterial blood pressure (MAP) of less than 60 mmHg, use of the NASG improved health outcomes by averting 2–3 disability-adjusted life years (DALYs) per woman and had a net savings or extremely low cost per DALY averted.

In 2012, the World Health Organization (WHO) recommended the NASG be used as a temporizing measure for women with PPH until definitive care, blood transfusions, and/or surgery can be obtained. Our aim was to evaluate the cost-effectiveness from the payer’s perspective of early NASG intervention using evidence from a cluster-randomized controlled trial of early NASG application at the primary health care (PHC) level prior to transport compared to later NASG application at the referral hospital (RH) level [5]. This study was conducted in Zambia and Zimbabwe, where the maternal mortality ratio is 591 and 960 per 100,000 live births respectively [14,15]. We used an econometric approach to evaluate the incremental cost-effectiveness of application of the NASG at the primary health care center (PHC) compared to waiting until the patient arrives at the RH. We hypothesized that early application of the NASG at the PHC would be more cost-effective than later application of the NASG at the RH.

## Methods

### Design of clinical study

The clinical data for this study was approved by the institutional review boards (IRBs) affiliated with the following institutions: University of California, San Francisco; University of Zambia, Lusaka; University of Zimbabwe-UCSF Collaborative Programme on Health Research; and the Department of Reproductive Health and Research of the World Health Organization. The cost data collected for this study did not involve human subjects, and was thus exempt from IRB oversight.

This cost-effectiveness analysis builds on a previously reported clinical trial, summarized here. The clinical trial data belongs to UCSF and is freely available with a UCSF data sharing agreement. The cluster-randomized controlled trial of 38 PHCs in Zimbabwe and Zambia enrolled patients from 2009 to 2012. Eligible PHCs were peri-urban with at least 500 annual deliveries that referred obstetric hemorrhage (OH) cases (≥500 mL blood loss) to one of five study regional hospitals. Participants were admitted at the PHC and were consenting women with any obstetric hemorrhage etiology and hypovolemic shock. Women with antepartum hemorrhage with a viable fetus were excluded. PHCs were randomized to either the early application or later application group using a covariate-constrained procedure to ensure balance across intervention arms on number of deliveries, number of deliveries per midwife, distance to referral hospital (RH), and proportion of OH cases expected [16].

Women who presented at the PHC at < 24 weeks gestation (ectopic pregnancy, complications of abortion, or trophoblastic/molar pregnancy) were enrolled in the outpatient department, while women at ≥ 24 weeks were enrolled in the maternity department. Midwives at the PHC were trained to treat PPH with uterotonics and IV fluids, repair first- and second-degree perineal lacerations, and refer any patient with estimated blood loss > 500 mL to the RH. PHCs were not equipped to provide blood transfusions, surgery, or manual vacuum aspiration (MVA). Each PHC had access to a shared ambulance system to transfer patients to the RH.

All eligible women had a perineal pad applied at study entry in the PHC to measure blood loss. Women in the early application arm received the NASG (Zoex Corporation, Coloma, CA 95613, USA) at the PHC and women in the later application arm received it at the RH per treatment protocol. All women were referred to the RH and were transported by ambulance, private vehicle, or taxi. Oxygen, IV fluids, uterotonics or uterine massage for uterine atony, suturing of lacerations, manual removal of placenta or retained tissues, MVA, surgery, and blood transfusions were available as needed at the RH. More detailed information regarding the design of the CRCT is provided elsewhere [5].

We use a per-protocol analysis [17]. Characteristics of women were similar between early and later NASG application groups (see Table 1) except for hemorrhage etiology. The early application group was composed of a higher proportion of women with uterine atony (42.1% vs. 28.7%) and a lower proportion with complications of abortion (15.6% vs. 36.2%) compared to the later application group.

**Table 1:** **Per**-**protocol study characteristics**

	**Early application**	**Later application**
	**n (%)**	**n (%)**
N° of women	366	466
Zambia	200	327
Zimbabwe	166	139
Mean age (standard deviation)	26.9 (5.9)	27.2 (6.3)
Median parity (IQR)	2 (1–3)	2 (1–3)
Gestational age (≥24 weeks)	37.7 (2.6)	37.4 (2.9)
Diagnosis		
Complications of abortion^***^	15.6%	36.2%
Postpartum uterine atony^***^	42.1%	28.7%
Retained placenta^*^	25.1%	19%
Lacerations/Genital trauma^**^	13.1%	7.5%
Placental abruption	0.8%	4.5%
Placenta previa	0.8%	1.1%
Ectopic pregnancy	0.5%	1.3%
Ruptured uterus	0.3%	1.2%
Placenta accreta^*^	1.4%	0.0%
Molar pregnancy	0.3%	0.4%
Median (IQR) estimated revealed blood loss at study entry (ml)	500 (480–700)	500 (500–800)

***p < 0.001, **p < 0.01, *p < 0.05.

Note: Wilcoxon Rank Sum test utilized to test all continuous variables due to non-normality. Chi-square test used for categorical values except where noted.

### Effectiveness

Disability-adjusted life years (DALYs) without age-weighting were used to quantify the burden of disease as a discounted sum of the number of years of life lost (YL) from early death and years lost due to disability (YLD) [18]. The timeframe of this analysis was the four-year period of the intervention. Disabilities over the women’s life were considered. YL was calculated as the difference between the woman’s age and her age-adjusted life expectancy within her country of residence for those women who died during the study. YLD was constructed as a composite of the morbidities for each woman who survived. This includes acute renal failure, acute respiratory distress syndrome, heart failure, cerebral impairment (seizures, unconsciousness, motor/cognitive loss), and severe anemia. The rate of severe anemia was defined as hemoglobin value less than 7.0 g/dL at hospital discharge.

There was no evidence of statistically significant differences between earlier and later NASG application across mortality and morbidity outcomes. The odds of death in the early application group were 64% lower (OR 0.36 (95% CI: 0.08 – 1.53) than the later application group (Table 2). There were no morbidities in the early application group and 0.2% in the later application group. There was no statistically significant difference in severe anemia at discharge between groups. As morbidities and mortalities were rare, there may have not been adequate statistical power to detect an effect [5]. There was no statistically significant difference between minutes from study entry to death or study exit between the two groups; however, women in the early application group recovered from shock at a significantly faster rate, 165 min for early application vs. 209 min for later application (OR 1.28 (95% CI: 1.05-1.57). On average, the later application group received the NASG 2.5 hours after the early application group.

**Table 2:** **Study treatments and outcomes**

	**Early application**	**Later application**	**Odds ratio (95% CI)**	**p**-**value**
Mortality	3/366 (0.8%)	12/466 (2.6%)	0.36 (0.08-1.53)	0.17
Zimbabwe	1/166 (0.6%)	1/139 (0.7%)		
Zambia	2/200 (1%)	11/327 (3.4%)		
Morbidity	0/363 (0.0%)	1/454 (0.2%)		
Anemic at discharge	74/321 (23.1%)	68/322 (26.8%)	1.10 (0.61 – 1.99)	0.74
Emergency hysterectomy	1/210 (0.4%)	0/295 (0.0%)		
Time variables (mean minutes)				
Study entry to death	282	391		
Study entry to shock recovery^α^	165 (90–279)	209 (114–386)	1.28 (1.05-1.57)^β^	0.015
Study entry to exit	608.1	608.8		
Study entry until NASG	2.4	144.1		

^α^Median (IQR); ^β^Hazard Ratio.

### Cost

We estimated costs using micro-costing methods. Resource use was estimated from clinical trial records. Unit costs were collected from pharmacies, blood banks, and hospital administrators in local currencies and converted into international dollars [19]. Costs of clinical resources at the PHC and RH were summed for each individual (Table 3). As the NASG was applied to both groups, only the timing differed; costs of the NASG (material/cleaning/training) were estimated and described below for reference only.

**Table 3:** **Unit costs by country**, **2010** (**IU**)^**1**^

**Costs**	**Zambia**	**Zimbabwe**
**1. NASG per use** ^**2**^	$1.54	$1.54
**2. Training per use** ^**3**^	$1.62	$1.62
**3. 10iu Oxytocin**	$0.20	$3.00
**4. 0.2 mg Ergometrine**	$0.20	$3.75
**5. Misoprostol**	$0.19	$0.81
**6. 1 unit of blood (450 ml)**	$42	$112.50^4^
**7. Emergency hysterectomy**	$36.56	$28.44

^1^International dollars.

^2^Cost is amortized over 72 uses and includes cleaning.

^3^Averaged across countries and includes provider opportunity cost.

^4^Mean of first 2 units shown; actual costs in analysis are $135 for first unit and $90 per each additional unit.

### Cost of the NASG

The material cost of the NASG per use was estimated as $1.04, based on an approximate price of $75 and an estimated life of 72 uses per garment (personal communication Neil McConnochie, BlueFuzion to Suellen Miller). The cost of cleaning the NASG included bleach, bucket for immersion, personal protection equipment, and personnel, and was estimated at $0.50 per use. The total estimate for cost of NASG was $1.54 per use.

Training costs included transportation, facilities, materials, and personnel costs. The model assumes training has a 10-year life, which is conservative given that training is not designed to require a refresher. Estimates of training cost per patient were based on actual costs collected from one facility where two hundred participants attended a stand-alone (NASG only) training. The base-case estimate for the cost of training per NASG use was $1.62.

### Clinical resource costs

Costs of uterotonics, oxytocin per ampoule (10 IU) and misoprostol per dose, were collected from hospital pharmacies and hospital administrators in one facility in Zambia and one facility in Zimbabwe. Cost of blood transfusions was based on cost per unit of blood in each country during the study period. The cost of one unit of blood and uterotonics in Zimbabwe were significantly higher than in Zambia. In Zimbabwe blood cost $135 for the first unit and $90 per each additional unit, compared to $42 per unit in Zambia. Blood was not always available during the study period.

Emergency hysterectomy (EH) costs for complications due to intractable uterine atony and complications of abortion were collected. Costs include personnel, equipment, anesthesia, and operating room costs. Emergency hysterectomies were conservatively estimated to require 6 personnel over 60 to 90 minutes. No other surgeries were included in this analysis, as etiologies differed, and some etiologies require surgery (ruptured uterus, ruptured ectopic pregnancy).

### Statistical methods

We estimated a series of models using Stata 13.0 (StataCorp, College Station, TX, USA). We anticipated that variances would differ across clusters due to variations in adherence to treatment protocol; we include random effects to allow individual-level differences to vary across clusters [20]. The general models for the random effects specifications are as follows:

$$ {E}_{ic} = {\beta}_0 + {\beta}_1{J}_c + {\beta}_2Zi{m}_c + {\varepsilon}_c + {\mu}_{ic}\kern0.72em \left[\mathrm{Effectiveness}:\ \mathrm{Models}\ 1 - 4\mathrm{a}\right] $$

$$ {C}_{ic} = {\delta}_0 + {\delta}_1{J}_c + {\delta}_2Zi{m}_c + {\varepsilon}_c + {\mu}_{ic}\kern0.48em \left[\mathrm{Cost}:\ \mathrm{Models}\ 5 - 8\mathrm{a}\right] $$

where *E*_*ic*_ is the probability of a disability-adjusted life year of individual *i* in cluster *c* and *C*_*ic*_ is the costs of individual *i* in cluster *c*; *J*_*c*_ is an intervention indicator for cluster *c* where j = 1 for early application group and j = 0 for later application group; *Zim*_*c*_ is an indicator for Zimbabwe (1 = Zimbabwe, 0 = Zambia) for each cluster *c*; and *u*_*ic*_ is the error term.

In order to determine *E*_*ic*_, the probability of a disability-adjusted life year of individual *i* in cluster *c*, we specified 4 random intercept models. In Model 1, the model was specified as above but excluded the country indicator. In Model 2, we added the country indicator. In Model 3, we estimated an interaction term between country indicator *Zim*_*c*_ and early application indicator *J*_*c*_ to understand country-specific effects of early application. In Model 4, we omitted the country indicator and instead used 4 referral hospital indicators, *RH*_*c*_, where the Lusaka hospital was the reference. We re-specified Model 4 for Zambia only (Model 4a) to improve statistical power as 13 of the 15 deaths occurred in Zambia. Models 5-8a were specified identical to Models 1-4a substituting cost of individual *i* in cluster *c* for outcome. We used a likelihood ratio test to compare model fit for Models 1–4 and 5–8.

We compared the effectiveness and costs between the earlier and later NASG application groups to calculate the incremental cost-effectiveness ratio (ICER) [21]. The ICER is the difference between the costs and effectiveness of the groups, given by *δ*_1_/*β*_1_.

We conducted a sensitivity analysis by simulating probabilistic clinical resource costs and mortalities to provide insight to their contribution to the ICER. We varied the unit cost of blood from $20 to $200 while keeping the blood transfusion rate (number of units per individual) and all other variables constant to reflect the probable range of costs of blood within sub-Saharan Africa. We also assessed how varying the relative odds ratio of death given timing of NASG application would impact the ICER, and whether the results from our primary models were consistent when stratifying by severe shock at study entry, defined by mean arterial pressure <60 mm Hg.

## Results

### Effectiveness

The model that fit the data best (Model 4) showed that women in the early application group had 0.712 fewer DALYs than those in the later application group (p < 0.05; Table 4). In the unadjusted model, the early application group experienced 0.38 DALYs and the later application group experienced 0.97 DALYs due to obstetric hemorrhage (not shown). Thus, early application averted 0.59 DALYs (95% CI: −1.58 to 0.39), although this difference was not statistically significant (Table 4). The relatively few deaths (n = 15) and morbidities (n = 1) in this trial precluded precise estimation of DALYs, demonstrated by the wide confidence intervals around each of the point estimates. Adding the country indicator for Zimbabwe to control for between-country variation (Model 2) and modeling an interaction effect between country and early application (Model 3) did not improve the fit of the model and the coefficient for the early application remained insignificantly different from zero. The best-fit model was Model 4, which accounted for referral hospital effects using the Lusaka hospital as reference. Women in the early application group averted 0.712 DALYs (p < 0.05) compared to those in the later application group. In Zambia only (Model 4a), we found a marginally significant effect (p < 0.10) of early application of 0.729 DALYs averted.

**Table 4:** **Random effects models for Disability**-**Adjusted Life Years (DALYs)**

	**(1)**	**(2)**	**(3)**	**(4)**	**(4a)** ^**3**^
Early application	−0.591 (0.51)	−0.553 (0.50)	−0.688 (0.624)	−0.712 (0.326)**	−0.729 (0.461)*
Zimbabwe^1^		−0.504 (0.55)	−0.670 (0.760)		
Zimbawe#EarlyApplication			0.425 (1.091)		
Lusaka^2^				ref	ref
Harare 1				0.112 (0.536)	
Harare 2				−0.595 (0.383)	
Copperbelt1				−0.204 (0.525)	0.203 (0.634)
Copperbelt2				−0.171 (0.511)	−0.166 (0.608)
Constant	0.967 (0.344)**	1.13 (0.390)**	1.188 (0.421)**	0.955 (0.262)***	0.961 (0.324)***
Country control	no	yes	yes	no	no
RH controls	no	no	no	yes	yes

***p < 0.01, **p < 0.05, *p < 0.1.

Note: Standard errors are in parentheses.

^1^Reference group is Zambia.

^2^RH include Lusaka (n = 341), Harare1 (n = 93), Harare2 (n = 211), Copperbelt1 (n = 82), Copperbelt2 (n = 95).

^3^Model 4a is the same as model 4, but restricted to Zambia only.

### Costs

#### Mean costs, by type

The mean treatment cost per woman across both groups was $51.17. The median cost was lower, at $19.61 (IQR $8.02 – $91.62), indicating that a small number of women had very high costs. The median cost was $19.62 (IQR $13.62- $25.62) in Zimbabwe and $8.82 ($8.02-92.02) in Zambia. The unadjusted mean cost per woman was similar across both countries at $54.56 for the early application group and $52.61 for the later application group (Table 5). Stratified by country, the treatment costs in Zimbabwe were $17.16 lower for early vs. later application (p = 0.275) and $15.51 higher for early vs. later application groups in Zambia (p = 0.001).

**Table 5:** **Costs and clinical resources**, **by NASG timing group and country**

**Costs and clinical resources**	**All women**			**Zimbabwe**			**Zambia**		
	**832 women in 38 clusters**			**305 women in 12 clusters**			**527 women in 12 clusters**		
	**Late**	**Early**	**p-value**	**Late**	**Early**	**p-value**	**Late**	**Early**	**p-value**
1. Number of women	466	366		139	166		327	200	
2. Proportion of women who received any blood transfusions	36.1%	27.9%		7.9%	5.8%		48.0%	61.0%	
3. Mean cost of blood transfusions (of those who received blood)	$114.88	$115.49	0.967	$500.91	$441.25	0.667	$87.83	$94.14	0.240
4. Proportion of women who received any uterotonics	78.8%	78.4%		82.0%	82.5%		77.4%	75.0%	
5. Mean cost of uterotonics (of those who received uterotonics)	$4.53	$7.42	<0.001	$13.46	$14.85	0.332	$0.52	$0.64	< 0.001
6. Mean cost per woman	$52.61	$54.56	0.765	$58.30	$41.14	0.275	$50.19	$65.70	< 0.001
	(43.6 - 61.6)	(45.6 - 63.5)		(31.3 - 85.2)	(23.9 - 58.3)		(44.2 - 56.2	(57.8 - 73.5)	

Note: Wilcoxon Rank Sum test utilized to test all continuous variables due to non-normality.

In Zimbabwe, the cost of a unit of blood was high and very few (7%) individuals received transfusions. In Zambia, roughly half of the women received blood transfusions and a slightly higher (statistically insignificant) proportion of women in the early application group received blood transfusion. Across both countries and intervention arms, three out of four women received an uterotonic; more than 97% of those receiving an uterotonic received oxytocin. In Zambia, costs of uterotonics are marginal. In Zimbabwe, costs per uterotonic dose were 4–15 times higher, although rates and costs did not differ meaningfully across intervention groups.

#### Cluster-specific random-effects models

Early application of the NASG cost $15.51 more than later application based on estimates from the best-fit model (Model 7). In our first cost model, treatment costs for the early application group were $1.96 higher on average than for the later application group, but this difference was not statistically significant (Table 6; Model 5). The difference in treatment costs across the intervention groups remained insignificant when controlling for country-specific costs (Model 6). Adding an interaction term between the country indicator and timing of application in Model 7 improved the fit of the model. We found a significant interaction effect between country and timing of application (Model 7), indicating the average treatment cost for the early application group in Zimbabwe was $32.67 less than the later application group (p < 0.01), while in Zambia, the mean treatment cost of the early application group was $15.51 higher (marginally significant, p < 0.1) than the later application group.

**Table 6:** **Random effects models for cost**

	**Model 5**	**Model 6**	**Model 7**	**Model 8**	**Model 8a** ^**3**^
Early application	1.957 (6.55)	3.14 (6.63)	15.51 (8.40)*	7.75 (6.99)	16.04 (4.96)***
Zimbabwe^1^		−7.63 (6.81)	8.81 (9.47)		
Zimbawe#EarlyApplication			−32.67 (13.64)***		
Lusaka^2^				ref	ref
Harare 1				−38.75 (11.5)***	
Harare 2				−9.52 (8.19)	
Copperbelt1				−36.31 (11.40)***	−35.84 (6.62)***
Copperbelt2				−19.93 (10.91)*	−21.92 (6.35)***
Constant	52.61 (4.16)***	54.88 (4.80)***	50.19 (5.17)***	63.34 (5.62)***	60.45 (3.38)***
Country control	no	yes	yes	no	no
RH controls	no	no	no	yes	yes

***p < 0.01, **p < 0.05, *p < 0.1.

Note: Standard errors are in parentheses.

^1^Reference group is Zambia.

^2^RH include Lusaka (n = 341), Harare1 (n = 93), Harare2 (n = 211), Copperbelt1 (n = 82), Copperbelt2 (n = 95).

^3^Model for Zambia only.

The above estimates may be biased if the availability and use of resources varied significantly across referral hospital. In Model 8, we substituted the country indicator with 4 referral hospital indicators (*RH*_*c*_) using the Lusaka hospital as reference and this improved the fit of the model compared to the simple model (Model 5). Compared to the Lusaka hospital, the Harare1, Copperbelt1, and Copperbelt2 hospitals had significantly lower treatment costs ($38.75, $36.31, and $19.93, respectively); however, there was no significant difference in cost by timing of application within this model. In Model 8a (restricted to Zambia), the mean treatment cost for the early application group was significantly higher than the later application group, at $76.49 vs. $60.45 respectively. Consistent with Model 8, Model 8a shows significantly lower treatment costs for the Copperbelt1 and Copperbelt2 hospitals compared to the Lusaka hospital.

### Incremental Cost-Effectiveness Ratio (ICER)

Across both countries, early NASG application costs $21.78 per DALY averted ($15.51/0.712) compared to late application based on the results from our best-fit models. In Zambia, early NASG application costs $22.00 for each DALY averted compared to late application ($16.04/0.729 DALYs). We do not have enough evidence to determine a statistically meaningful result for Zimbabwe.

### Sensitivity analyses

First, to assess for potential non-adherence to treatment protocol that might affect a per-protocol analysis, we ran the same models using an intent-to-treat (ITT) analysis. There were no significant differences in effects between the approaches; the estimates of the per-protocol analysis were attenuated slightly by the smaller sample size, as expected. The low attrition rate and negligible degree of bias suggest that our per-protocol results are what we would expect in a clinical practice setting.

We reran Models 1–8, stratified by shock status. Women who entered the study with mean arterial blood pressure of less than 60 are considered to be in severe shock. There were no differences in estimates in our cost models. However, the DALYs averted rose from 0.712 to 0.844 in the best-fitting effectiveness model. This suggests that, in this sample, early application of the NASG before transport appeared to have greater benefit for women who did not enter in severe shock.

**Figure 2:**
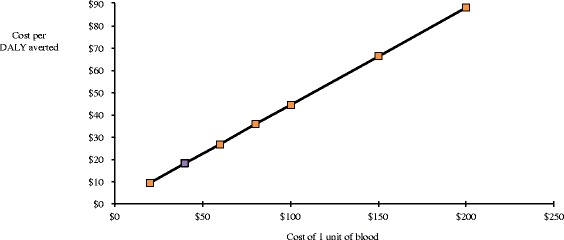
**Sensitivity analysis of cost of 1 unit of blood.** The cost per unit of blood causes the ICER to increase because there was a slightly higher blood transfusion rate in the early application group. The base-case value is indicated in purple.

**Figure 3:**
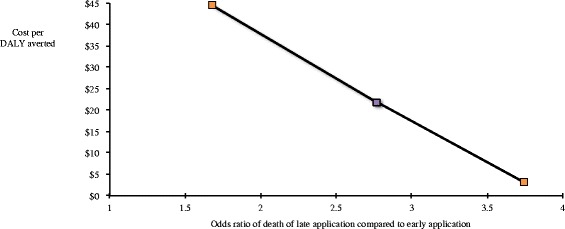
**Sensitivity analysis of effectiveness.** The cost per DALY averted falls as the odds of death in the late application group increases relative to the odds of death in the early application group. The base-case value is indicated in purple.

The ICER of applying the NASG early compared to later was sensitive to the unit cost of blood transfusions and ranged from $9.22-87.85 (see Figure 2). In this case, it appeared less cost-effective when the cost of blood increased because there was a slightly higher blood transfusion rate in the early application group. The baseline odds ratio of death with the late application of NASG is 2.77 times higher than early application. We imputed deaths in the timing groups to create synthetic odds ratios ranging from 1.68 to 3.74, which were selected based on the expected effectiveness. The ICER falls as odds of death in the early application group decreases relative to the group with later application (Figure 3). In other words, as early application becomes more effective, it becomes more cost-effective.

## Discussion

Within a cluster randomized trial, early application of the NASG at the primary care level versus waiting until referral hospital arrival had a cost-effectiveness ratio of $21.78 per DALY averted ($15.51 in added costs divided by 0.712 DALYs averted per woman, both statistically significant). In Zambia, early NASG application cost a nearly identical $22.00 for each DALY averted compared to late application ($16.04/0.729 DALYs). This ratio is considered “very cost-effective” by the WHO definition of annual GDP per capita [22]. In Zimbabwe, point estimate results suggested similar benefits, but were not statistically significant, which may be due to low statistical power from the few events within this site.

Mean treatment costs were patterned differently by country, where the early application group was associated with somewhat lower but not statistically different costs in Zimbabwe ($17.16), but significantly higher costs ($15.51) in Zambia in our descriptive analyses, and consistent with the random-effects Model 7.

The difference in costs across the two countries is largely driven by the rate and cost of blood transfusion (Figure 2). This is not surprising given that blood transfusion is the definitive treatment for hypovolemia. In Zimbabwe, very few women received blood (5.8% and 7.9%, respectively in the early and late application groups) compared to Zambia (61.0% and 48.0%, respectively). Some of this difference may be explained by the higher per unit cost of blood in Zimbabwe compared to Zambia ($113 vs. $42). We are not able to directly quantify the extent to which blood availability contributed to this difference in rates. However, our analysis that took into account referral hospital (RH) effects revealed higher costs in the Lusaka RH versus the Copperbelt RHs. Given that the Lusaka RH is co-located with the national blood bank and has better access to blood, we suspect this is likely due to higher proportions of women at the Lusaka RH receiving blood transfusions. Local blood availability and costs should be contextualized to the country and region as lack of blood is common in low- and middle-income contexts and is a major driver of hemorrhage-related mortality and morbidity [23].

We found that mild shock (MAP > = 60) on presentation was associated with more DALYs averted per women who received the NASG earlier. This may be due to the considerable benefit of early application of the NASG to *prevent* worse shock during transport to definitive treatment. Importantly, this finding is inconsistent with previous results from multiple studies, which found greater NASG benefits for women in severe shock when applied at the RH level only [24]. Therefore, we found NASG having important value for women in severe and mild shock.

Results from the previous cost-effectiveness analysis on data from the referral hospital level, which compared NASG application to no application, determined the intervention to be cost saving (Egypt) or extremely cost-effective (Nigeria at $3.13 per DALY averted) [13]. As expected, our current analysis of data from Zambia and Zimbabwe of early NASG application at the PHCs compared to later NASG application at the RH indicates that the additional costs associated with expanding the NASG to the PHC level is well within the WHO standards for very cost-effective [25]. Our country-level findings suggest that NASG intervention at the PHC may even be cost saving in Zimbabwe; however, the low rate of mortalities and morbidities in that subsample renders the findings imprecise. Although the results of the cluster-randomized trial lacked statistical significance, the large observed reduction in mortality (64%) was consistent with statistically significant outcomes from earlier studies conducted at the RH level [6,7]. Furthermore, earlier NASG intervention in the CRCT was significantly associated with faster recovery from shock, which supports the plausibility of a meaningful reduction in mortality (Table 2).

Policy makers with limited resources may need to make decisions about which levels of the health care system to place the NASG. Should NASG be only at the RH? At both the RH and the PHC? Or should it be used at even lower levels of the health care continuum? Should NASGs be on ambulances or other transport?

Given the strength of the evidence for cost-effectiveness at the referral hospital level, the decision to implement the NASG within the RH level is straightforward. In deciding whether to incur the additional costs of adding the NASG at the PHC level, policy makers may want to consider several factors: 1) the proportion of women who first seek care at the PHC level; 2) the proportion of maternal deaths from hemorrhage which occur at the PHC, during transit, or within the first 2 hours after admission to the RH; 3) the clinical capacity for resuscitation at the PHC level; 4) average transport times from the PHC to the RH; and 5) the length of delays in admission and receipt of quality definitive care including blood transfusions and surgery once at the RH. Greater benefits will be obtained from situating the NASG at the PHC level in contexts where the first contact with care is at the PHC level, or where receipt of definitive care is delayed by transportation, less optimal resuscitation or other time delays faced at the PHC or RH. Data on situating the NASG within emergency transport are not available.

There are several limitations to this study. The major limitation was low power due to a smaller than expected number of deaths in the CRCT. Also, the study was not designed to explicitly study the availability and provision of blood transfusion; thus, we had less reliable information about the availability of blood transfusions. Given that the earlier and later application arms occurred simultaneously and blood transfusions do not appear to be clustered within any set of patients referred from a PHC, we expect non-differential bias across intervention arms.

The analysis did not include the economic benefits and costs from the patient’s perspective. It is likely that early application of the NASG would have health and economic benefits for women and their families.

The costs for using the NASG will fluctuate, but may continue to decrease, as competitive sales pricing has resulted in prices as low as $57.50. (Personal communication Neil McConnochie, Blue Fuzion to Suellen Miller.) Incorporating training into ongoing Emergency Obstetric Newborn Care, Life Saving Skills, and pre-service training will eventually eliminate a separate price for stand-alone in-service NASG training. The differential costs for blood, medications, and surgery will be highly context specific.

## Conclusion

Applying the NASG earlier at the Primary Health Care level for women in hypovolemic shock secondary to obstetric hemorrhage instead of waiting until the woman has been transferred to the Referral Hospital has the potential to be a cost-effective decision across many clinical settings. Our evidence from Zambia strongly supports this conclusion. Evidence from Zimbabwe is suggestive but not statistically significant. As the NASG is designed to reverse shock and decrease further bleeding for women in obstetric hemorrhage, patients who have received the NASG earlier may be better able to survive delays in reaching definitive care at the RH and recover more quickly from shock, all at a cost that is highly acceptable. Policymakers and administrators need to consider a variety of factors to determine which levels of their health system will cost-effectively save the most lives.

## Abbreviations

NASG: Non-pneumatic Anti-Shock Garment
RH: Referral hospital
PHC: Primary Health Center
DALY: Disability-adjusted life year
MAP: Mean arterial blood pressure
WHO: World Health Organization
OH: Obstetric hemorrhage
PPH: Post-partum hemorrhage
YL: Years of years of life lost
YLD: Years lost due to disability
CRCT: Cluster-randomized clinical trial
MVA: Manual vacuum aspiration
ICER: Incremental cost-effectiveness ratio
